# Ursolic Acid Protects Neurons in Temporal Lobe Epilepsy and Cognitive Impairment by Repressing Inflammation and Oxidation

**DOI:** 10.3389/fphar.2022.877898

**Published:** 2022-05-16

**Authors:** Kun-mei Liu, Yue Huang, Pan-pan Wan, Yun-hua Lu, Ning Zhou, Juan-juan Li, Chun-yang Yu, Jin-jiang Chou, Lianxiang Zhang, Chun Zhang, Yuan-yuan Qiang, Rui Zhang, Le Guo

**Affiliations:** ^1^ Department of Microbiology and Biochemical Pharmacy, School of Pharmacy, Ningxia Medical University, Yinchuan, China; ^2^ Ningxia Key Laboratory of Cerebrocranial Disease, Ningxia Medical University, Yinchuan, China; ^3^ Medical Science Research Institution of Ningxia Hui Autonomous Region, Ningxia Medical University, Yinchuan, China; ^4^ College of Life Sciences, Huzhou University, Huzhou, China; ^5^ Experimental and Molecular Pathology, Institute of Pathology, Ludwig-Maximilians-University, Munich, Germany; ^6^ Ningxia Key Laboratory of Clinical and Pathogenic Microbiology, General Hospital of Ningxia Medical University, Yinchuan, China; ^7^ Department of Medical Laboratory, School of Clinical Medicine, Ningxia Medical University, Yinchuan, China

**Keywords:** temporal lobe epilepsy, cognitive impairment, ursolic acid, anti-inflammatory, antioxidant, neuroprotective

## Abstract

Temporal lobe epilepsy (TLE) is characterized as an impaired ability of learning and memory with periodic and unpredictable seizures. Status epilepticus (SE) is one of the main causes of TLE. Neuroinflammation and oxidative stress are directly involved in epileptogenesis and neurodegeneration, promoting chronic epilepsy and cognitive deficit. Previous studies have shown that ursolic acid (UA) represses inflammation and oxidative stress, contributing to neuroprotection. Herein, we demonstrated that UA treatment alleviated seizure behavior and cognitive impairment induced by epilepsy. Moreover, UA treatment rescued hippocampal neuronal damage, aberrant neurogenesis, and ectopic migration, which are commonly accompanied by epilepsy occurrence. Our study also demonstrated that UA treatment remarkably suppressed the SE-induced neuroinflammation, evidenced by activated microglial cells and decreased inflammation factors, including TNF-α and IL-1β. Likewise, the expression levels of oxidative stress damage markers and oxidative phosphorylation (OXPHOS) enzyme complexes of mitochondria were also remarkably downregulated following the UA treatment, suggesting that UA suppressed the damage caused by the high oxidative stress and the defect mitochondrial function induced by SE. Furthermore, UA treatment attenuated GABAergic interneuron loss. In summary, our study clarified the notable anti-seizure and neuroprotective properties of UA in pilocarpine-induced epileptic rats, which is mainly achieved by abilities of anti-inflammation and anti-oxidation. Our study indicates the potential advantage of UA application in ameliorating epileptic sequelae.

## Introduction

Temporal lobe epilepsy (TLE) is the most common form of partial epilepsy characterized by impaired learning and memory abilities. Periodic and unpredictable partial complex seizures occur in this condition (Mikulecká et al., 2019). Current anti-seizure drugs (ASDs) mainly function *via* blocking the voltage-gated channels and modulating the ion channels. Repression of neurotransmission and attenuation of the excitatory neurotransmission are also involved in the mechanisms of ASDs function (Akyüz et al., 2021; Turner and Perry, 2017). Although the effect of ASDs is clear on seizure cessation, they still fail to improve the pathophysiological scores after epilepsy or prevent TLE progression specifically (Sills and Rogawski, 2020). Moreover, some ASDs aggravate the synaptic injury and cognitive disability (Cramer et al., 2010). Hence, it is necessary to discover new drugs with fewer side effects.

TLE is mainly caused by status epilepticus (SE) (Devinsky, 2004), which is defined as continuous seizure activity over 30 min or consecutive seizures without consciousness recovery (Trinka et al., 2012). SE causes hippocampus damage by inducing inflammation, neuronal damage, and insufficient GABA inhibition. The hippocampus damage finally results in chronic TLE and cognitive impairment. However, the neurons damage and cognitive impairment induced by epilepsy are ameliorated through the anti-inflammatory effect. In addition, the loss of GABAergic neurons, aberrant neurogenesis, and abnormal migration of newborn dentate granule cells from the SGZ to GCL induced by epilepsy are eased *via* repression of inflammation facilitated (Long et al., 2017; Wang et al., 2019). Meanwhile, elevated oxidative stress also persistently exists in the entire development of epilepsy and contributes to mitochondrial dysfunction, neuronal damage, and cognitive impairment (Rowley and Patel, 2013). Therefore, it is a strategy to attenuate seizure activity and improve cognitive function by suppressing the inflammatory response and oxidative stress induced by SE.

UA is a type of pentacyclic triterpenoid compound in the form of free acid or saponins aglycone. UA exists in numerous classes of medicinal plants, such as *Eriobotrya japonica* and *Hedyotis diffusa*. The wax coating of several fruits also contains UA (Tuli et al., 2013). UA exhibits a wide spectrum of important biological activities of cancer suppression, anti-diabetes, anti-bacteria, anti-hyperlipidemia, hepatoprotection, and immunomodulation (Kashyap et al., 2016). In addition, previous studies clarify that UA protects neurons by alleviating oxidative stress and inflammation in neurodegenerative diseases, including cerebral ischemia, psychosis, anxiety, depression, Alzheimer’s disease (AD), and Parkinson’s disease (PD) (Habtemariam and longevity, 2019). UA inhibits NF-кB translocation to the nucleus by restraining the MAPK signaling pathway, which reduces the secretion of inflammatory mediators and represses the activation of microglia (Teismann et al., 2004). Moreover, reactive oxygen species (ROS) formation is also inhibited by UA, contributing to the protection of neurons (Ramos-Hryb et al., 2017). Notably, GABAergic interneurons are frequently lost during SE, leading to insufficient inhibition of GABA (Sun et al., 2007). Studies show that the GABA level is elevated by UA by confining the activity of GABA transaminase (GABA-T) (Ibarra et al., 2010). Nevertheless, the effect of UA on epileptogenesis and cognitive disability induced by lithium pilocarpine remains elusive.

In this study, we evaluated the effect of UA on seizure susceptibility, inflammation, oxidation, nerve regeneration, and cognitive dysfunction in epileptic rats induced by lithium-pilocarpine. Our study investigated the protective role and mechanism of UA in epilepsy and cognitive impairment, providing a promising drug candidate for the new anti-epileptic drug development.

## Materials and Methods

### Animals

Male Sprague–Dawley (SD) rats (180–220 g) were purchased from the Laboratory Animal Center of Ningxia Medical University [Ningxia, China, permit number: SCXK (Ning) 2020-0001]. The rats were acclimatized per cage under standard laboratory conditions of temperature (23 ± 1°C) and humidity (40%–60%) for about 1 week by keeping a 12 h light/12 h dark cycle (lighted from 8:00 a.m. to 8:00 p.m.). Prior to the experiments, rats had free access to food and water. All animal experiments were approved by the Ethics Committee of Ningxia Medical University (no. 2018-094) and performed according to the guidelines of the Chinese National Institutes of Health.

### Lithium-Pilocarpine Model and Experiment Design

**FIGURE 1:**
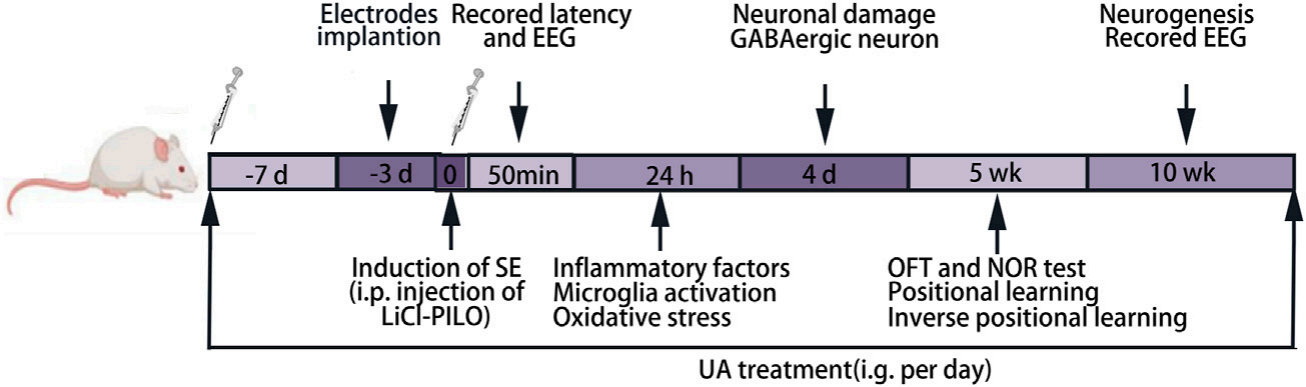
Experimental timeline. LiCl-PILO: lithium-pilocarpine; OFT: open-field test; NOR: novel object recognition.

A total of 126 rats were randomly divided into four groups. In the normal control group (NC group), animals were treated with the respective vehicles only. Animals in the model group (SE group) were treated with lithium chloride-pilocarpine. Regarding previous investigations, 20–25 mg kg^−1^ d^−1^ UA seems to be an effective dose in neurological disorders (Rai et al., 2016; Wang et al., 2011), and high doses appear to have a cytotoxic effect (Supplementary Figure S1). In the present study, the animals in two administration groups respectively received UA (Sigma, St. Louis, MI, United States, suspended in 0.1% aqueous solution of Tween 80) at doses of 20 mg kg^−1^ d^−1^ (UA20 group) and 100 mg kg^−1^ d^−1^ (UA100 group) by intragastric administration 7 days prior to lithium chloride-pilocarpine treatment, also given after SE induction until the whole experiment ended. The animals receiving valproic acid (Rhawn, Shanghai, China) treatment at a dose of 200 mg kg^−1^ d^−1^ instead of UA were positive control (VPA group). The procedure of the lithium-pilocarpine-induced epileptic the rat model was carried out as follows: animals were injected intraperitoneally (i.p.) with lithium chloride (LiCl; Damao, Tianjin, China) at a dose of 127 mg kg^−1^ at 18–20 h before pilocarpine (30 mg kg^−1^; Sigma, United States) injection. Before 30 min of pilocarpine injection, 1 mg kg^−1^ atropine (i.p.; Sigma, United States) was injected to decrease peripheral cholinergic side effects induced by pilocarpine. The behavioral alteration of rats was monitored after injection with pilocarpine. The seizure severity was assessed using the Racine scale (Racine et al., 1972). Rats with epileptic seizures of stage Ⅳ were selected for further experiments. After 1 h of SE induction by pilocarpine injection, 6% chloral hydrate (5 ml kg^−1^, i.p.; Shanpu, Shanghai, China) was injected to attenuate seizure activity and reduce mortality. The timeline of the experiments is indicated in Figure 1.

### Seizure Susceptibility and Video-Encephalographic (Video-EEG) Analysis

Epileptic seizures were observed within 1 h after the occurrence of SE (*n* = 6 rats per group). Seizure scores were assessed based on the Racine scale. Latency of developing seizures and percentages of SE were recorded to evaluate the effect of UA on seizure susceptibility. After anesthesia, rats of each group were fixed to a stereotactic device. Burr holes were made in the skull, and then two screws linked to recording electrodes were placed bilaterally on the dura mater of the frontoparietal cortex to implant EEG recording electrodes. Another stainless steel screw was implanted in the cerebellar region to fix electrodes with dental cement. The SE model was established after a three-day recovery. After pilocarpine injection, EEG data were recorded immediately for 50 min using the HF-12 mobile function experimental platform (Chengdu Taimeng Software Co., Ltd., China) and analyzed using the BL-420 software. In addition, after 10 weeks of SE induction, EEG analyses were also performed for 7 days to evaluate spontaneous seizures in the chronic phase.

### Positional Learning and Position Reversal Learning

After 5 weeks of SE induction by pilocarpine, the IntelliCage (TSE Systems GmbH, Germany) was used to automatically monitor the rats’ abilities of spontaneous positional learning and positional reversal learning by setting different experimental procedures (Wu et al., 2017) (*n* = 6 rats per group). The cage of IntelliCage is equipped with four intelligent learning conditioning chambers. There are two drinking bottles in each cage, and the cage with a small transponder-reader antenna that can register the entering rats with a microchip. The rats trigger sensors by nosepoking or visiting to open the doors and drink water freely. As each rat is implanted with a unique microchip, the corner entry and licking of the water bottle nozzle data are automatically recorded by the system. During the whole experiment, UA was intragastrically administrated once at 8:30 a.m. each day. We set up three IntelliCage learning modules. In the free exploration module, all doors could be open to access the water bottle and for free access to food and water. Rats were allowed to be familiar with the environment for 7 days. In the position learning module, 7 days was needed totally. In this module, the rat’s least preferred corner during the free exploration period was designated as the “correct” corner, while other corners were designated as “error” corners. Only the “correct” corner was reached, and then the door was opened for visiting, nosepoking, and linking. Similarly, positional reversal learning was also performed for 7 days. In this module, the new “correct” corner was opposite to the “correct” corner in the position learning stage, and other corners were designated as “error” corners. The learning ability was evaluated by the number of visits, nosepokes, and links of the “correct” corner.

### Open-Field Test and Novel Object Recognition

After 5 weeks of SE induction, an open-field test (OFT) and novel object recognition (NOR) test were performed to assess motor ability and memory ability (*n* = 6 rats per group). For OFT, the movements of each rat in an open-field arena (1 m × 1 m × 30 cm) without any object were recorded for 10 min. The total distance was calculated to determine the motor ability (Jiang et al., 2020). In the NOR test, three stages included habituation for 5 min, training for 10 min, and testing for 10 min. The habituation stage aimed to reduce the stress and anxiety caused by an unfamiliar environment. At this stage, animals were allowed to explore the open-field arena freely without any objects for 5 min. At the training stage, animals were allowed to explore two identical objects in the arena within 10 min. At the testing stage, one of the objects was replaced by a similar size novel object with a different shape, and rats were allowed to explore freely for 10 min. To eliminate the influence of residual odor from the previous rat in the test, 75% ethanol solution was used to clean the arena and objects before the next stage started. The Smart 3.0 (Panlab Co., Spain) software was used to track and record the time spent and the distance traveled. Rats with a total exploring time ≤15 s, spending more than 75% of the time on one object during training, or only exploring one object in the testing stage were excluded from the data analysis (Bellantuono et al., 2020). The recognition index (RI) of a familiar object or novel object for each animal was calculated y using the formula: RI = TA or TB/(TA + TB) (TA = time spent exploring the familiar object A; TB = time spent exploring the novel object B).

### Brain Tissue Processing

The hippocampal tissues of rats were quickly isolated and collected on ice. Tissues were weighed and stored at −80°C for ELISA and Western blot assay. After being anesthetized, rats had transcardial perfusion with 4% paraformaldehyde (PFA), and brain tissues were removed and fixed with 4% PFA overnight. Then, brain tissues were dehydrated with 20% and 30% sucrose water. Finally, the 20 μm sections were obtained with a cryostat and collected serially in 12-well plates containing phosphate buffer. The frozen sections were fixed on slices and dried at room temperature for experiments or stored at −80°C for Nissl, FJC, and immunofluorescence staining.

### Nissl and Fluoro-Jade® C Staining

The slices of brain tissues at 4 days after SE were washed with PBS for 10 min. For Nissl staining, sections were stained with crystal violet solution at 56°C for 30 min, followed by washing with distilled water. Subsequently, the slices were differentiated in differentiation solution until the backgrounds were nearly colorless. 70%, 80%, 95%, and 100% of ethanol were used successively to dehydrate and clear with xylene. Finally, the slices were sealed with neutral gum and imaged by optical microscopy (Nikon, Tokyo, Japan). The procedures for FJC staining were described by Wang et al. (2008). Briefly, following treatment with an alcohol-sodium hydroxide mixture, 0.06% potassium permanganate was used. Then, an FJC working solution was used for FJC staining. After drying in darkness at 50°C –60°C, the slices were cleared with xylene in the dark, and then cover slipped with DPX. Finally, FJC-stained brain sections were observed under a fluorescence microscope (Leica VS 120, Wetzlar, Germany), and the images of interest were representatively captured (3 rats per group, two sections per rat, and two random non-overlapping fields per section were analyzed).

### Immunofluorescence

The slices of brain tissues at 24 h, 4 days, or 10 weeks after SE induction were washed with PBS for 10 min and heated in citrate buffer to retrieve antigen for 20 min. The slices were permeabilized with 0.3% Triton X-100 three times (5 min each), and then the sections were incubated with the following primary antibodies: anti-CD68 (1:500, Abcam); anti-somatostatin (1:500, Abcam); anti-parvalbumin (1:500, Abcam); anti-NeuN (1:500, Abcam); anti-neuropeptide Y (1:500, Abcam); anti-PROX1 (1:500, Abcam); anti-doublecortin (1:200, Abcam); and anti-Reelin (1:500, GeneTex) at 4°C overnight after blocking with 1% BSA for 1 h. Washing with PBST 3 times, the slices were incubated with Alexa Fluor^®^ 488 (1:1000, Abcam) and Alexa Fluor^®^ 647 (1:1000, Abcam) at room temperature over 2 h. DAPI was used for nuclear staining, and the images were observed and collected by a fluorescence microscope (Leica VS 120, Wetzlar, Germany) (3 rats per group, two sections per rat, and two random non-overlapping fields per section were analyzed).

### Estimation of TNF-α, IL-1β, 8-OHdG, and Malondialdehyde

The supernatant of hippocampal tissues homogenate was extracted at 24 h after SE to detect the expression level of cytokines. For estimating the expression levels of TNF-α and IL-1β, ELISA kits (Elabscience, Wuhan, China) were used according to the protocol provided by the manufacturer. Briefly, after adding standard or sample solution to each well, biotinylated detection Ab was added and incubated. Following HRP conjugate incubation, the substrate reagent was added. Subsequently, a stop solution was added, and plates were read at 450 nm in a microplate reader (Bio-Tek, PowerWaveTMXS) immediately. Hippocampal 8-hydroxy-2-deoxyguanosine (8-OHdG) expression level was determined using an 8-OHdG ELISA kit (Elabscience, Wuhan, China). According to the manufacturer’s instructions, a standard or sample solution was added to each well, and biotinylated detection Ab was added immediately. Then, the substrate reagent was added after incubating with HRP conjugate. Read plates immediately after adding the stop solution. Quantification of the malondialdehyde (MDA) level in the hippocampus was achieved using the rat MDA colorimetric assay kit (Solarbio, Beijing, China). Distilled water was served as a blank control. Samples were added to other tubes. After adding the MDA test solution, thiobarbituric acid solution was added. After mixing and heating in a boiling water bath at 100°C for 60 min, the supernatants of mixtures were taken and the absorbance was measured at 450, 530, and 600 nm (*n* = 6 rats per group).

### Assay of Inflammatory Cytokine

Rat Cytokine ELISA Plate Array (EA-4004, Signosis, United States) was used for evaluating inflammatory cytokines. According to the manufacturer’s protocol, the concentration of total protein from the homogenate of rats’ hippocampal tissues was normalized to 0.3 μg μl^−1^. Briefly, 100 μl diluted samples were added to each well, and then the plate was covered and incubated for 2 h at room temperature with gentle shaking. The plate was washed with 200 μl of 1 × Assay wash buffer three times, and then 100 μl of diluted biotin-labeled antibody mixture was incubated with samples. After washing with wash buffer three times, 100 μl diluted streptavidin-HRP conjugate was added. Subsequently, a 95 μl freshly prepared substrate solution was added after washing with wash buffer three times (10 min each time). Finally, the plate was read by a luminometer after incubating for 2 min (*n* = 6 rats per group).

### Western Blot Assay

The hippocampal tissue was homogenized in lysis buffer containing protease inhibitor (KeyGen, Jiangsu, China) and centrifuged at 12,000 g for 5 min at 4°C to extract the total protein. The supernatant was collected, and the total protein concentration was determined using the BCA assay kit (KeyGen, Jiangsu, China). Subsequently, proteins (30 μg) were loaded in 10% SDS-PAGE gels to separate according to their molecular weights and transferred to PVDF membranes. The membranes were blocked for 1 h with 1% BSA diluted in TBST at room temperature and then incubated with Total OXPHOS Rodent WB Antibody Cocktail (1:1000, Abcam) at 4°C overnight. After washing three times (5 min each time) with TBST, the membranes were incubated with HRP-conjugated Goat Anti-Mouse IgG (1:2000, Abbkine) at room temperature for 1 h. β-Actin expression was served as an internal reference, and the signals were quantified by densitometry analysis using ImageJ software (*n* = 3 rats per group).

### Statistical Analysis

Data were presented as mean ±SD (*n* ≥ 3). All statistical analysis and graph illustrations were performed using GraphPad Prism 8.0. One- or two-way ANOVA with post hoc Dunnett’s test was performed to indicate significant differences. For the novel object recognition test, paired *t*-test was performed. *p* < 0.05 was considered statistically significant.

## Results

### Ursolic Acid Ameliorated Seizure Susceptibility and Inhibited Chronic Epilepsy Development

**FIGURE 2:**
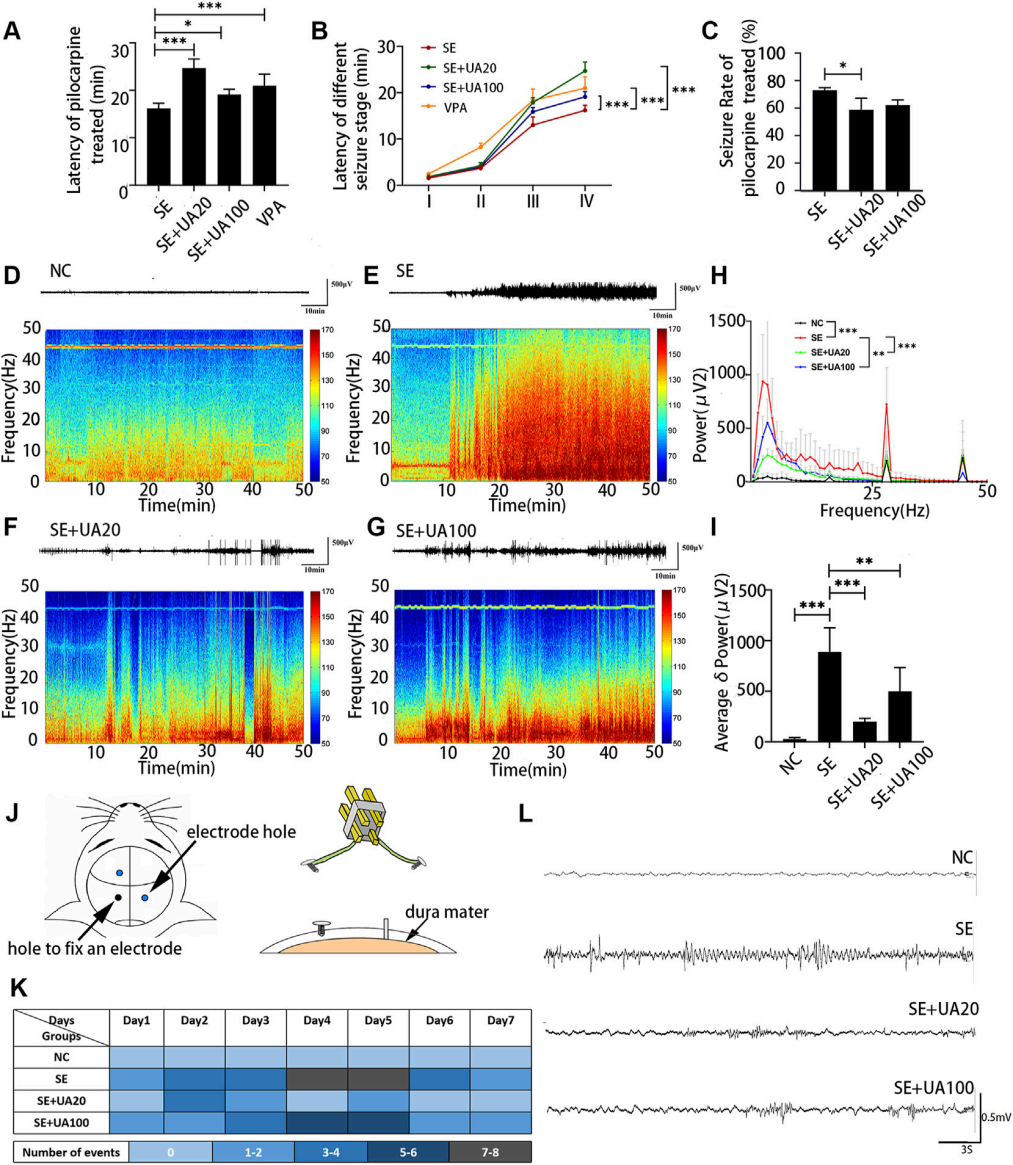
UA reduced the seizure susceptibility and inhibited chronic epilepsy development of the model. **(A)** The latency of seizure occurrence induced by lithium-pilocarpine. **(B)** The latency of different seizure stage induced by lithium-pilocarpine. **(C)** Percentages of rats with developed SE induced by pilocarpine. **(D–G)** Representative traces of EEG activity and spectrograms of representative EEG recordings for 50 min. Bar: 500 μv, 10 min. **(H)** FFT analysis of EEG recordings. **(I)** Comparison of the average δ (0.75–4 Hz) power. **(J)** Diagram of electrode implantation. **(K)** Number of “EEG seizure” events in each group. Each individual box was color-coded according to the legend. Each of the numbers in these boxes indicated the total number of events per day. **(L)** Example of events in each group. Bar: 500 μv, 3 s (*n* = 6, all values are expressed as means ±SD, ^∗∗∗^ *p* < 0.001, ^∗∗^ *p* < 0.01, ^∗^ *p* < 0.05 *vs*. SE, one-way ANOVA, or two-way ANOVA).

In the process of generating two epileptic models induced by lithium chloride-pilocarpine and pentylenetetrazol (Supplementary Figure S2), UA treatment efficiently ameliorated the induced seizure behavior and prolonged the onset of SE compared to the SE group (Figure 2A, *p*_UA20_ < 0.0001, *p*_UA100_ = 0.0224). Furthermore, UA treatment with 20 and 100 mg kg^−1^ d^−1^ dosages delayed the onset of different seizure stages (Figure 2B, *p*_ua20_ < 0.0001, *p*_UA100_ = 0.0002). However, only 20 mg kg^−1^ d^−1^ dosage remarkably decreased the percentage of rats (*p* = 0.0329) with developed SE induced by pilocarpine (Figure 2C). It indicated that UA has a notable anti-seizure effect.

To explore correlated phenotypes, we performed intracranial cortical electroencephalography (EEG) and filmed simultaneous videos for rats in each group. Fast-Fourier transform (FFT) analysis of EEG data (Figures 2D–H) showed that the specific frequency band (0–50 Hz) power of the SE group was notably increased compared to the NC group (*p* < 0.0001). Conversely, the frequency band power was reduced in the UA20 group and UA100 group compared to the SE group (P_UA20_ = 0.0007, P_UA100_ = 0.0062). It was also confirmed by the heat maps of frequency band power. In addition, the average *δ* (0.75–4 Hz) power was markedly elevated in the SE group compared to the NC group (*p* < 0.0001), but it was reversed by UA treatment (Figure 2I, P_UA20_ < 0.0001, P_UA100_ = 0.0018). Digram of electrode implantation is indicated in Figure 2J. After 10 weeks of SE induction, we observed that epileptic rats exhibited spontaneous seizures, especially during cage changing. Compared to the UA20 group and UA100 group, spontaneous seizures and Ictal epileptic EEG abnormalities in the SE group were observed more frequently (Figures 2K–L). The above results suggested that UA treatment not only ameliorated the seizure susceptibility of pilocarpine-treated rats but also reduced the frequency of spontaneous seizures during the chronic phase of epilepsy.

### Ursolic Acid Ameliorated Cognition Deficit Induced by Epilepsy

**FIGURE 3:**
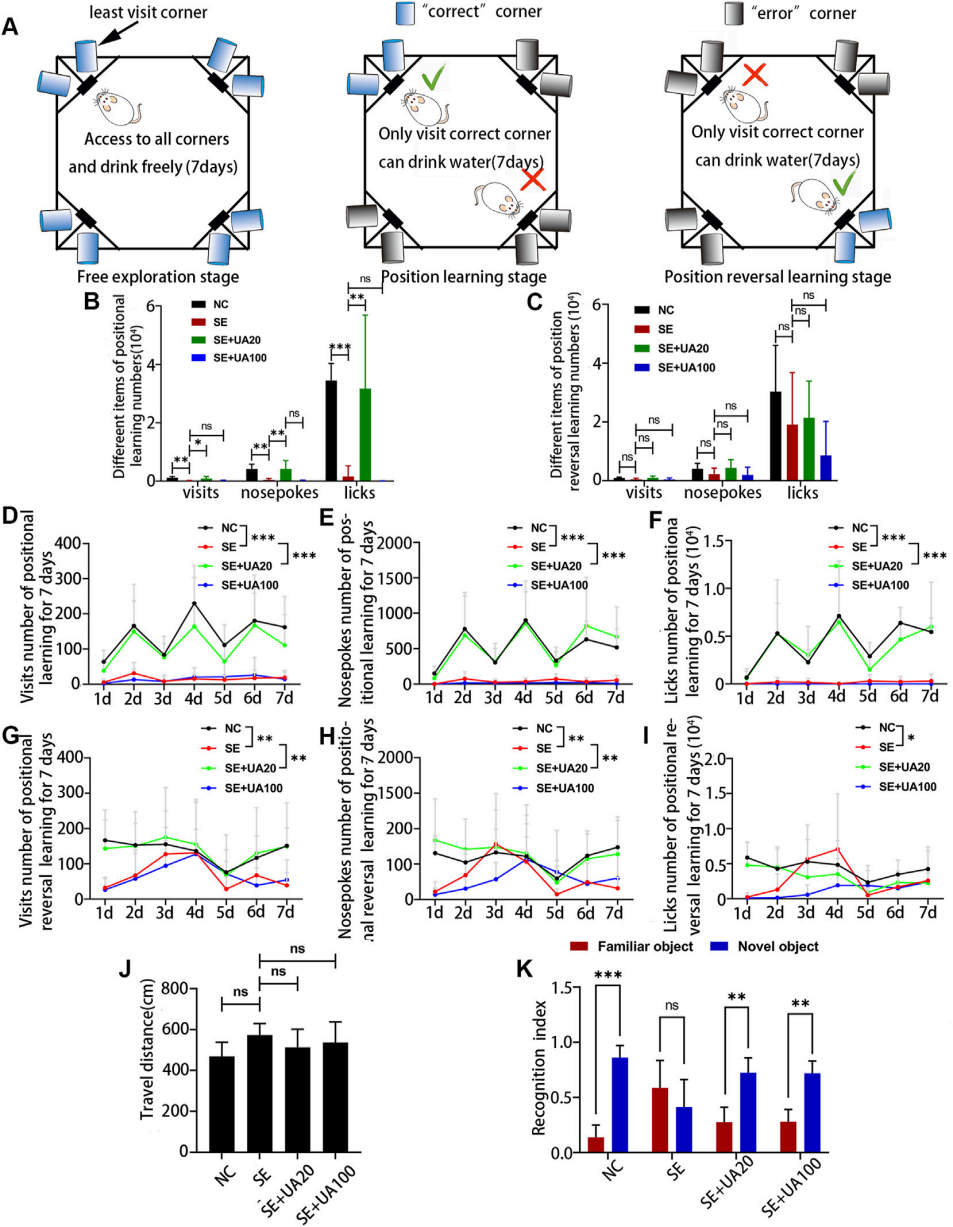
UA improved cognitive dysfunction induced by epilepsy. **(A)** Architectural diagram of IntelliCage and experimental program settings. Cage was not drawn to scale. **(B)** The number of different items of positional learning stage and **(C)** positional reversal learning stage. Number of visits **(D)**, nosepokes **(E),** and licks **(F)** in positional learning from d 1 to d 7. Number of visits **(G)**, nosepokes **(H),** and licks **(I)** in positional reversal learning from d 1 to d 7. **(J)** Open-field test of rats with developed SE induced by pilocarpine at 5 weeks. **(K)** Novel object recognition test of rats with developed SE induced by pilocarpine at 5 weeks. (*n* = 6, all values were expressed as means ±SD, ^∗∗∗^ *p* < 0.001, ^∗∗^ *p* < 0.01, ^∗^ *p* < 0.05 *vs*. SE, one-way ANOVA, two-way ANOVA, or paired *t*-test).

Because UA had been identified to have a notable effect of anti-seizure, we investigated the impact of UA on cognition deficit using IntelliCage (Figure 3A). The results of positional learning (Figure 3B) suggested that the number of visits, nosepokes, and licks was obviously lower in the SE group than in the NC group. These numbers were increased in the UA20 group. However, no difference was observed between the SE group and the UA100 group. The results of positional reversal learning (Figure 3C) suggested no differences in the SE group compared to the UA treatment groups, as well as compared to the NC group. We also assessed the number of visits, nosepokes, and licks from d 1 to d 7, respectively. The results indicated that the number of visits, nosepokes, and licks was lower in the SE group during the positional learning stage, compared to the NC group (Figures 3D–F, *p*_visits_ < 0.0001, *p*_nosepokes_ < 0.0001, *p*_licks_ < 0.0001) and UA20 group (*p*_visits_ < 0.0001, *p*_nosepokes_ < 0.0001, *p*_licks_ < 0.0001). However, only slight changes were recorded between the UA100 and the SE group (*p*_visits_ > 0.9999, *p*_nosepokes_ = 0.9408, *p*_licks_ = 0.9788). Similarly, the number of visits, nosepokes, and licks was also lower in the SE group compared to the NC group (*p*_visits_ = 0.0022, *p*_nosepokes_ = 0.0084, *p*_licks_ = 0.0345) in the positional reversal learning stage (Figures 3G–I). The number of nosepokes and visits was increased in the UA20 group compared to the SE group (*p*_visits_ = 0.0015, *p*_nosepokes_ = 0.0019), but the difference in licks number is insignificant (*p* = 0.9150).

Subsequently, we performed the OFT and NOR test on memory because of the less stress on the tested animals (Jiang et al., 2020). Locomotor activity was evaluated by OFT (Figure 3J), and the results indicated insignificant differences between each group during 10 min. In the NOR test (Figure 3K), rats failed to distinguish between novel objects and familiar objects in the SE group (*p* = 0.4348), whereas rats were able to differentiate between novel objects and familiar objects in the UA20 group (*p* = 0.0097) and UA100 group (*p* = 0.0046). Thus, the impaired ability to recognize novel objects was indifferent to the motor ability. Collectively, UA treatment ameliorated cognition deficit induced by epilepsy, but the UA100 group did not appear to improve spatial cognition deficit.

### The Neuroprotective Effect of Ursolic Acid

**FIGURE 4:**
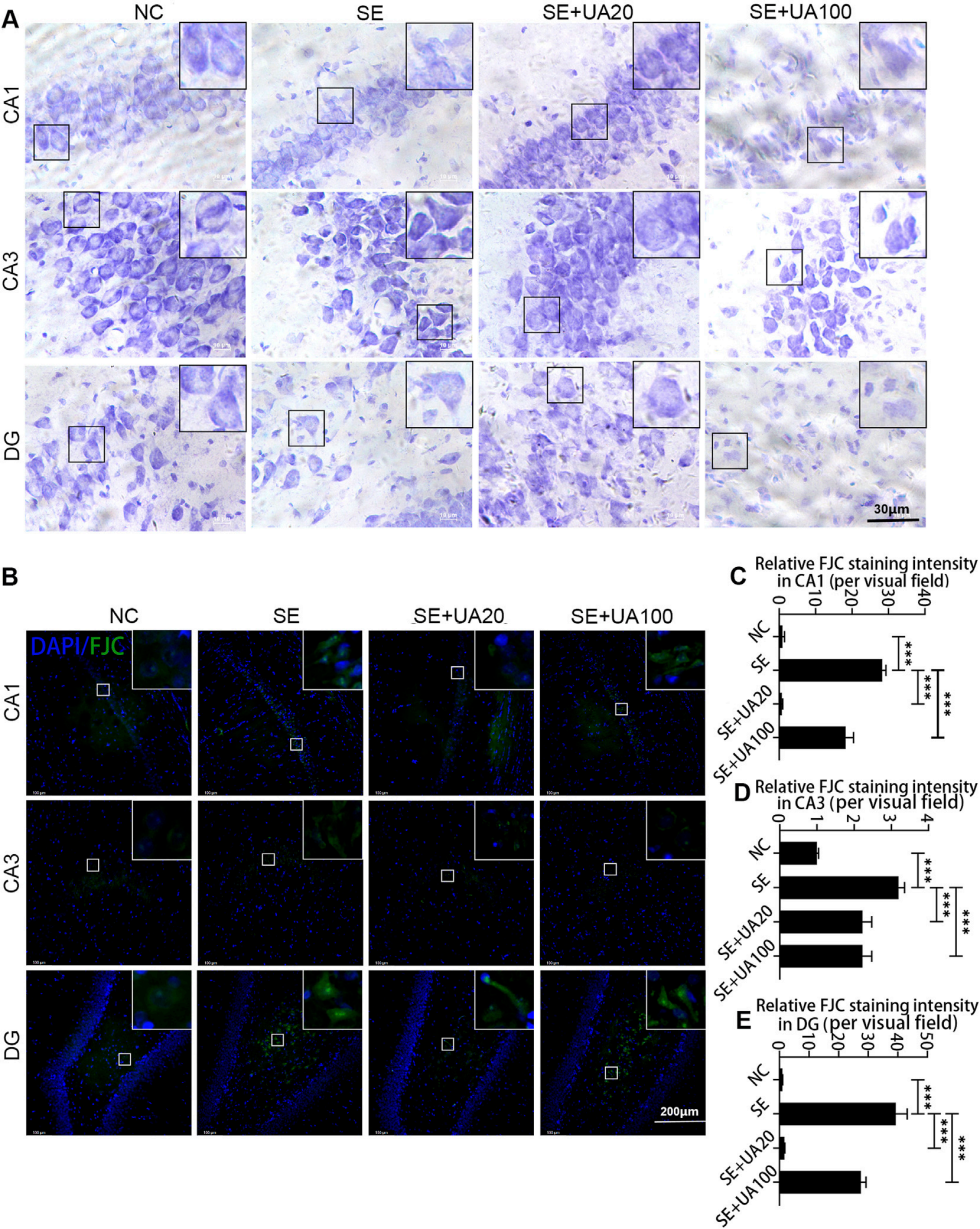
The neuroprotective effect of UA during SE. Nissl **(A)** and FJC **(B)** staining were used to detect the damage and repair of hippocampal neurons in the CA1, CA3, and DG regions of each group of rats. Relative staining intensities of FJC in CA1 **(C)**, CA3 **(D),** and DG **(E)** were compared (three rats per group, all values are expressed as means ±SD, ^∗∗∗^ *p* < 0.001 *vs*. SE, one-way ANOVA).

Neuronal injury is strongly associated with SE occurrence (Alsaegh et al., 2021; Zhu et al., 2020). Therefore, we evaluated the effect of UA on hippocampal neuronal injury by Nissl staining (Figure 4A) and FJC staining on d 4 after SE induction (Figures 4B–E). Intact morphology and clear contours were observed in the CA1/CA3 neuron and DG areas in the NC group. The neuron was shrunk, and the arrangement was loose in the SE group. In contrast, the injury of the hippocampal neuron was rescued, represented by clearer contours and tight arrangement in the UA20 group. Unexpectedly, the results of the UA100 group were comparable to the SE group. Moreover, the FJC staining intensity was increased dramatically in CA1 (*p* < 0.0001) and DG (*p* < 0.0001) in the SE group compared to the NC group. In contrast, SE-induced neuroapoptosis in the hippocampus was rescued by UA treatment. In summary, UA treatment notably reduced the neuronal damage induced by SE in each hippocampus area.

### Ursolic Acid Promoted Neurogenesis in Epilepsy

**FIGURE 5:**
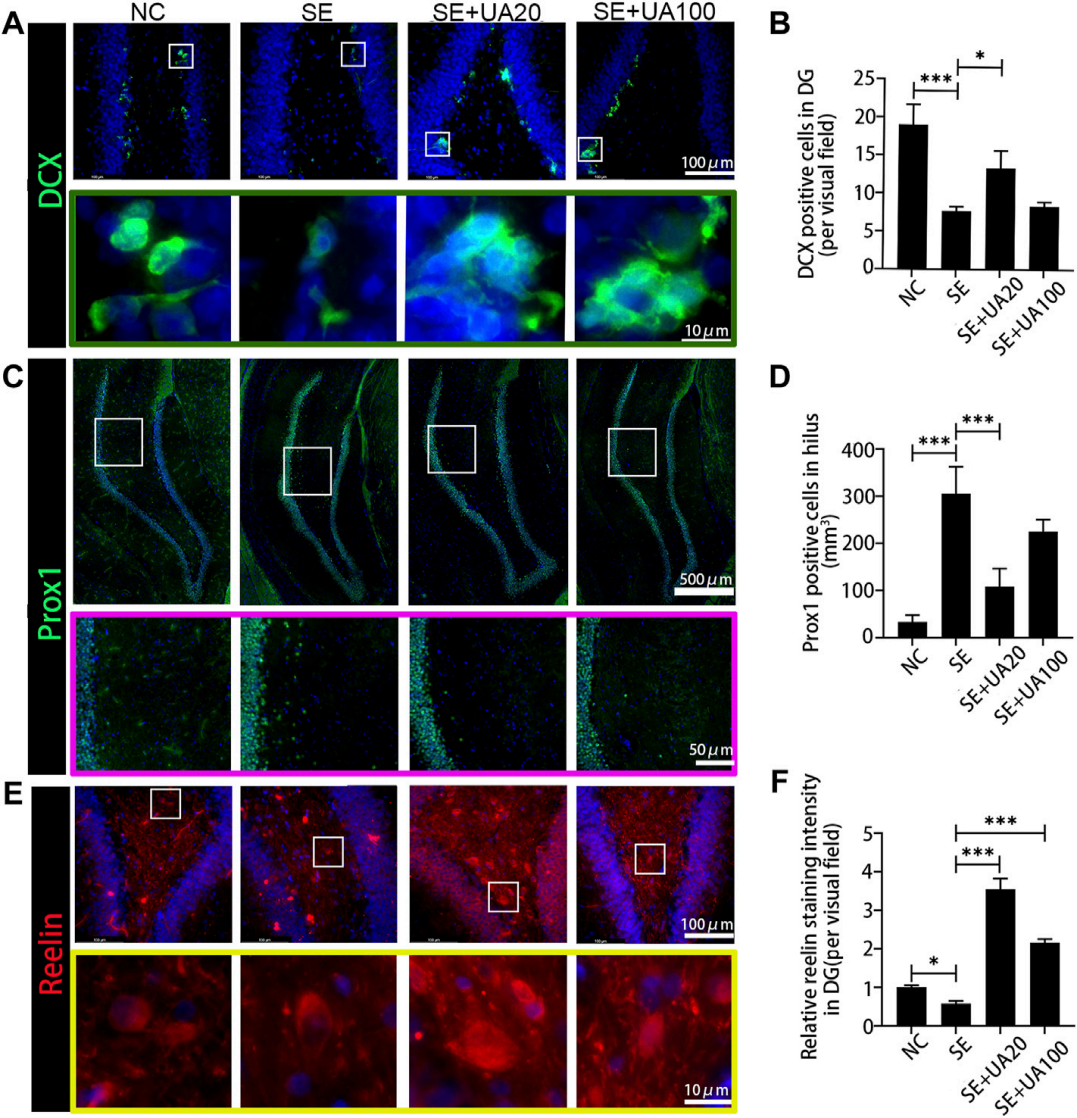
UA enhanced the neurogenesis and attenuated the ectopic migration 10 weeks after SE induction. **(A)** The immunofluorescence staining for DCX^+^ (green) in the DG area. Nuclei were counterstained with DAPI (blue). **(B)** Number of DCX^+^ cells in the DG area. **(C)** Granule cells were immunolabeled with the granule cell-specific marker Prox1 (green) in the DG area. Nuclei were counterstained with DAPI (blue). **(D)** Number of Prox1^+^ cells in hilus. **(E)** The immunofluorescence staining for reelin^+^ interneurons (red) in the DG area. Nuclei were counterstained with DAPI (blue). **(F)** Relative staining intensity of Reelin in DG area of the hippocampus after SE induction (three rats per group, all values are expressed as means ±SD, ^∗∗∗^ *p* < 0.001, ^∗^ *p* < 0.05 *vs*. SE, one-way ANOVA).

To evaluate the impact of UA treatment on neurogenesis, we detected the expression of DCX, a marker of newborn neurons, after 10 weeks of SE induction (Figures 5A,B). The results showed that DCX^+^ cells clustered along the subgranular zone (SGZ), and the number of DCX^+^ cells was reduced in the SE group compared to the NC group (*p* < 0.0001). Conversely, UA20 treatment increased the number of DCX^+^ cells (*p* = 0.0005). Furthermore, the granule cell layer in the NC group expressed more Prox1 thoroughly, which is a critical factor in DG development. Prox1^+^ cells were enriched in the hilus after 10 weeks of SE induction (*p* < 0.0001), while UA treatment largely abrogated this effect (Figures 5C,D, *p* < 0.0001). Reelin is an extracellular protein involved in synaptic homeostasis, which affects the shapes, sizes, and types of dendritic spines. Reelin dysregulation occurs in epileptogenesis (Liu, et al., 2018). The immunofluorescence results revealed obvious repression of reelin in rat hippocampal DG regions in the SE group compared to the NC group (*p* = 0.0234). Conversely, reelin was validated to be substantially upregulated following the UA treatment compared to the SE group (*p*_UA20_ < 0.0001, *p*_UA100_ < 0.0001). Interestingly, the level of reelin was even higher in DG regions with UA treatment compared to the NC group (Figures 5E,F). Collectively, the above results demonstrated that UA promoted neurogenesis and reduced neuronal ectopic migration in epilepsy.

### Ursolic Acid Attenuated Neuroinflammation Induced by Status Epilepticus in the Hippocampus

**FIGURE 6:**
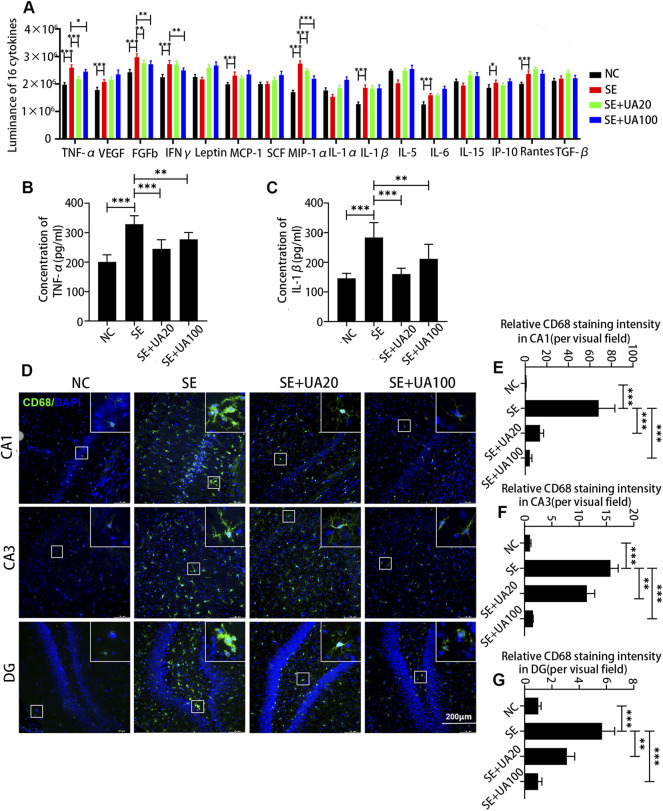
UA attenuated neuroinflammation induced by SE in the hippocampus. **(A)** The luminance of 16 inflammatory cytokines, *n* = 6. The expressions of TNF-α **(B)** and IL-1β **(C)** at different doses or vehicles, *n* = 6. **(D)** Immunofluorescence staining of activated microglia in the CA1, CA3, and DG regions of the hippocampus. Cell nuclei were stained by DAPI (blue). The activated microglia marked by CD68 were visualized with Alexa Fluor^®^ 488 secondary antibodies (green). **(E–G)** Diagrams showed the relative staining intensity of CD68 in the CA1, CA3, and DG areas of the hippocampus, three rats per group. (^∗∗∗^ *p* < 0.001, ^∗∗^ *p* < 0.01 *vs.* SE, all values are expressed as means ±SD, one-way ANOVA).

Neuroinflammation induced by SE is an important factor leading to nerve injury (Terrone et al., 2019). We detected 16 inflammatory cytokines in the hippocampus after 24 h of SE induction *via* ELISA assay (Figure 6A). Compared to the NC group, the expression levels of TNF-ɑ, VEGF, FGFB, IFNγ, MCP-1, MIP-1ɑ, IL-1β, IL-6, IP-10, and Rantes were markedly elevated in the SE group. Conversely, the UA treatment largely repressed the expressions of TNF-α, FGFB, IFNγ, and MIP-1α. In addition, TNF-α and IL-1β, which are closely related to the activation of microglia, were further quantified by ELISA assay due to the intermolecular interference and the inaccuracy of luminance (Hiragi et al., 2018). The results suggested that both TNF-α (*p* < 0.0001) and IL-1β (*p* < 0.0001) were increased in the SE group compared to the NC group (Figures 6B,C). In the UA treatment group, attenuated TNF-α (*p*_UA20_ < 0.0001, *p*_UA100_ = 0.0094) and IL-1β (*p*_UA20_ < 0.0001, *p*_UA100_ = 0.0091) levels were observed. Therefore, the UA treatment suppressed the excessive release of neuroinflammatory factors induced by SE.

Microglia activation has been reported to play an important role in mediating cytokine expression in the neuronal injury of the hippocampus during the SE (Rizzi et al., 2003). To confirm the inhibitory effect of UA on microglia activation, we detected the expression of CD68 (a marker of activated microglia) after 24 h of SE induction (Figures 6D–G). Microglia was highly activated in CA1, CA3, and DG areas in the SE group compared to the NC group, evidenced by upregulated CD68 (*p* < 0.0001). Enlarged and aggregated somas were also observed in the SE group. However, UA treatment largely abrogated the effect of SE induction on CD68 (*p*_UA20_ < 0.0001, *p*_UA100_ < 0.0001). Somas were smaller and showed a fusiform or branched shape with UA treatment. Hence, UA attenuated neuroinflammation by inhibiting microglia activation.

### Ursolic Acid Reduced Oxidative Stress Response Induced by Status Epilepticus in the Hippocampus

**FIGURE 7:**
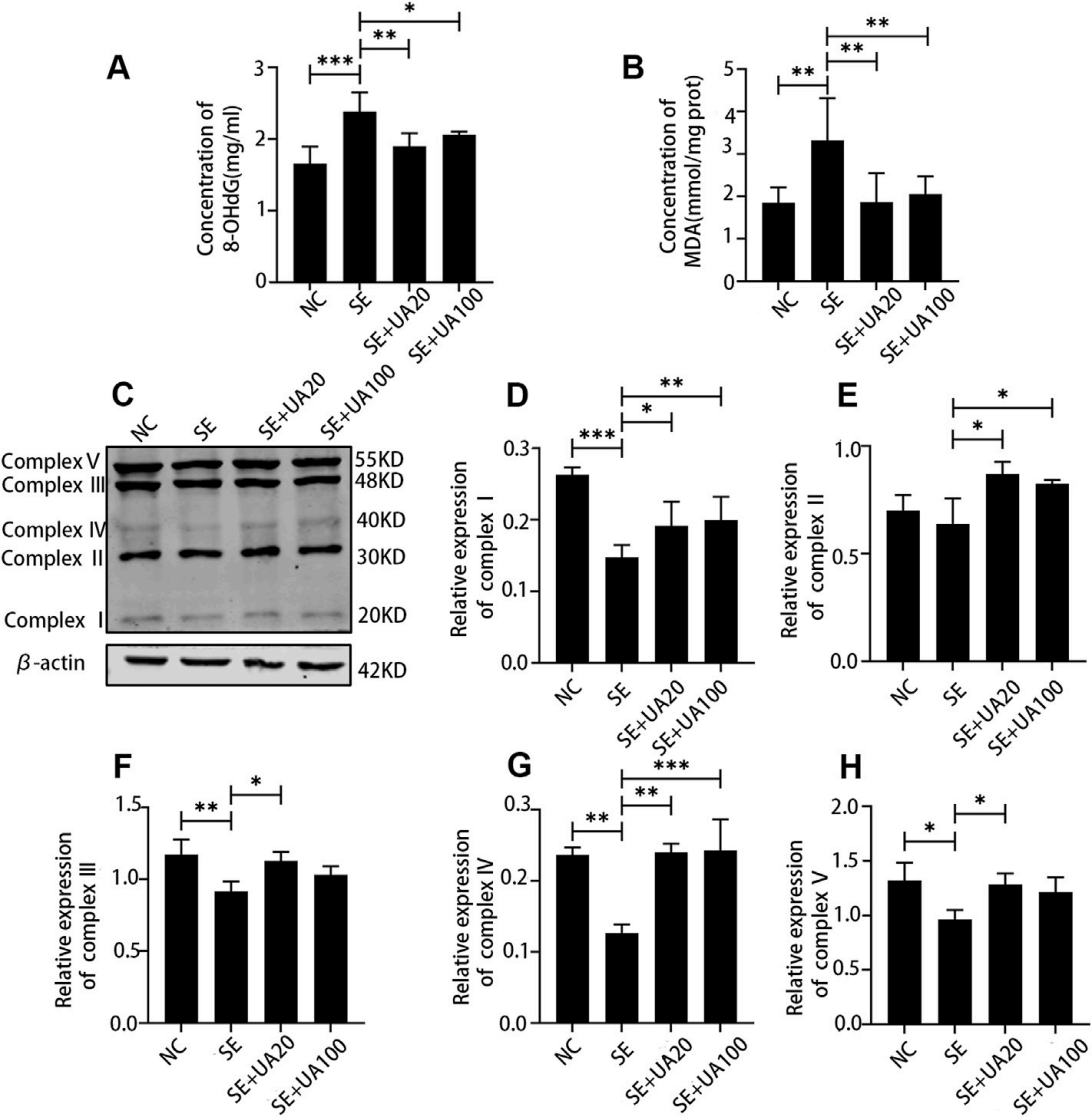
UA reduced oxidative stress and improved the mitochondrial function induced by SE in the hippocampus. The expression of 8-OHdG **(A)** and MDA **(B)**, *n* = 6. **(C)** Western blot assay of oxidative phosphorylation (OXPHOS) enzyme complexes of mitochondria. **(D–H)** Relative density analysis of the OXPHOS enzyme complexes I–V of mitochondria. β-Actin was probed as an internal control, *n* = 3. (^∗∗∗^ *p* < 0.001, ^∗∗^ *p* < 0.01, ^∗^ *p* < 0.05 *vs*. SE, all values were expressed as means ±SD, one-way ANOVA).

Next, we analyzed the effect of UA on oxidative stress response at 4 days after SE. Compared to the NC group, SE induction promoted the expression of 8-OHdG (Figure 7A, *p* < 0.0001), which is a marker of oxidative damage for mtDNA. High 8-OHdG expression induced by SE was reversed in the UA20 and UA100 groups (*p*_UA20_ = 0.0014, *p*_UA100_ = 0.0308). Because MDA is an index of lipid peroxidation (Haznedar et al., 2019), we assessed the MDA level *via* colorimetric assay (Figure 7B). The result showed that MDA concentration was increased in the hippocampus of the SE group compared to the NC group (*p* = 0.0028). By contrast, UA treatment resulted in an obvious reduction of hippocampal MAD level compared to the SE group (*p*_UA20_ = 0.0032, *p*_UA100_ = 0.0099). As mitochondrial dysfunction is related to oxidative stress, we also detected the expressions of oxidative phosphorylation (OXPHOS) enzyme complexes in mitochondria (Figures 7C–H). Compared to the NC group, the expressions of OXPHOS complexes were globally reduced in the SE group, except for complex II (*p* = 0.6402). In particular, complexes I and IV were repressed utmost in the SE group (P_I_ < 0.0001, P_III_ = 0.0013). UA treatment largely enhanced OXPHOS complex expressions compared to the SE group. Taken together, our results indicated that UA enhanced mitochondrial function and inhibited oxidative stress response during SE.

### Ursolic Acid Inhibited the GABAergic Neuron Damage Induced by Status Epilepticus

**FIGURE 8:**
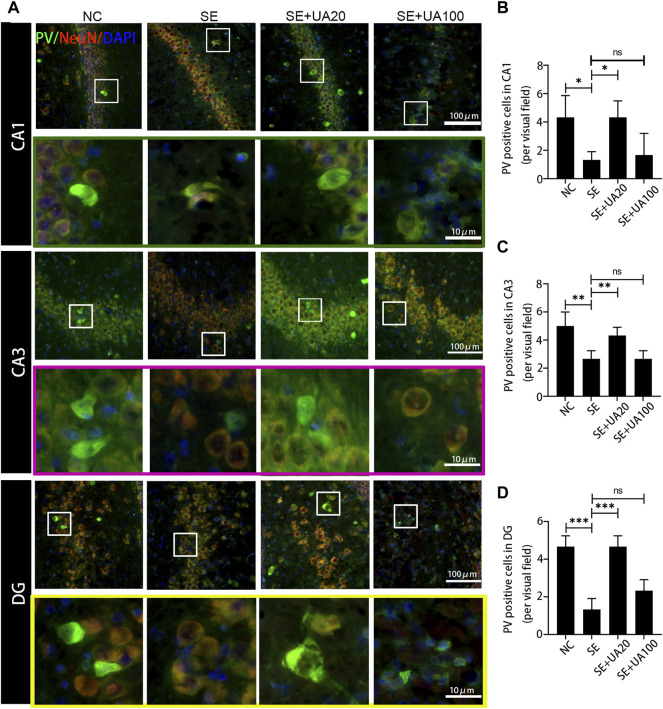
UA promoted the PV expression after SE induction. **(A)** Immunofluorescent triple-staining of PV (green), NeuN (red), and DAPI (blue) in the CA1, CA3, and DG regions of rat hippocampus. **(B–D)** The number of PV^+^ cells in each region of the hippocampus after SE induction. (three rats per group, all values were expressed as means ±SD, ^∗∗∗^ *p* < 0.001, ^∗∗^ *p* < 0.01, ^∗^ *p* < 0.05 *vs*. SE, one-way ANOVA).

**FIGURE 9:**
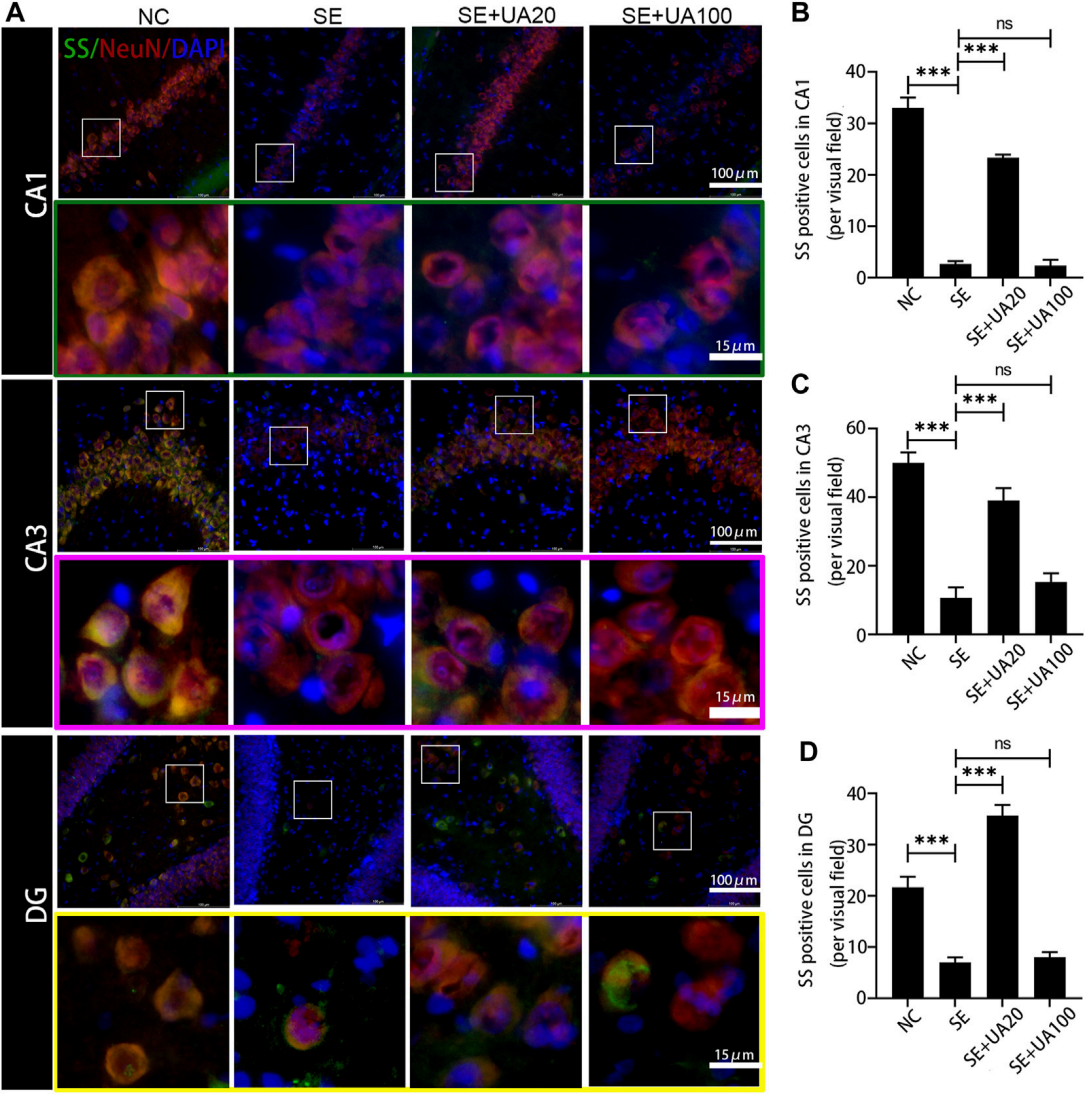
UA promoted the SS expression after SE induction. **(A)** Immunofluorescent triple-staining of SS (green), NeuN (red), and DAPI (blue) in the CA1, CA3, and DG regions of rat hippocampus. **(B–D)** The number of SS^+^ cells in each region of hippocampus after SE induction (three rats per group, all values were expressed as means ±SD, ^∗∗∗^ *p* < 0.001, ^∗^ *p* < 0.05 vs. SE, one-way ANOVA).

**FIGURE 10:**
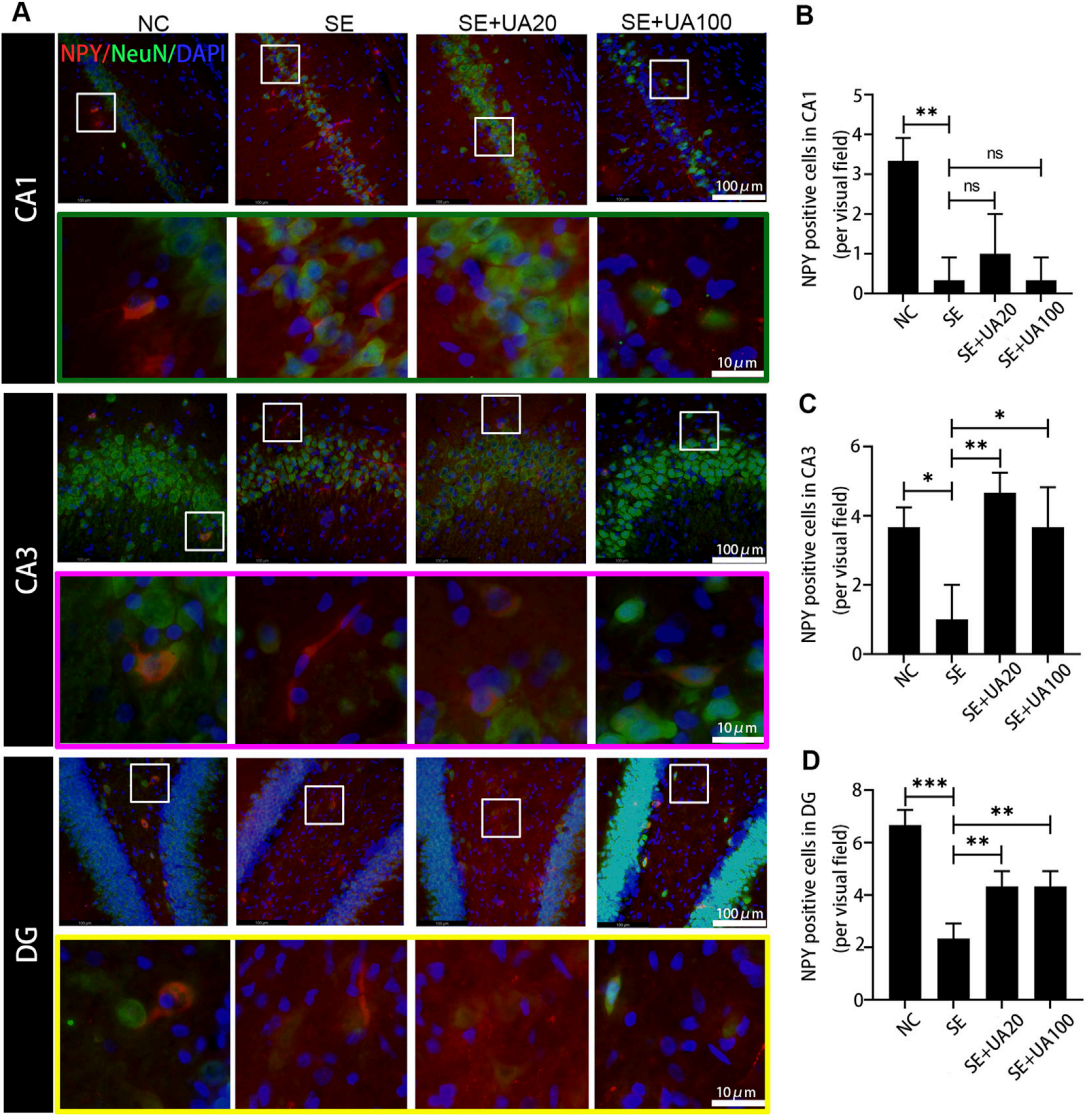
UA promoted the NPY expression after SE induction. **(A)** Representative images of immunofluorescent triple-staining of NPY (red), NeuN (green), and DAPI (blue) in the CA1, CA3, and DG regions of rat hippocampus. **(B–D)** The number of NPY^+^ cells in each region of the hippocampus after SE induction (three rats per group, all values were expressed as means ±SD, ^∗∗∗^ *p* < 0.001, ^∗∗^ *p* < 0.01 *vs*. SE, one-way ANOVA).

Because epilepsy accompanies GABAergic neuron loss and damage (Shetty and Upadhya, 2016), we investigated the effect of UA on inhibiting GABAergic neuron damage caused by SE. The distribution of the GABAergic interneuron was examined in the CA1, CA3, and DG regions (Figures 8–10). Three GABAergic interneuron markers (PV, SS, and NPY) and a neuronal marker (NeuN) were applied in double-labeled immunofluorescence staining. In the NC group, most NeuN^+^ neurons and fractional basket cells expressed PV. Tremendous PV^+^ cell bodies were clearly observed in the DG, CA1, and CA3 regions. In contrast, fewer PV^+^ interneurons were found in these regions in the SE group. In the UA20 group, the number of PV^+^ cells was increased compared to the SE group. The difference between the UA100 and the SE groups was unobvious (Figures 8A–D). In the CA1 and CA3 regions, the expression of SS was mainly focused in NeuN^+^ cells. Some basket cells also expressed SS in the DG region (Figures 9A–D). In addition, the impaired SS expression in each region during the seizure was largely rescued in the UA20 group, whereas the effect was unremarkable in the UA100 group in CA1 and DG regions. Similarly, the number of NPY^+^ cells in CA1, CA3, and DG of the SE group was decreased compared to the NC group (*p*_CA1_ = 0.0012, *p*_CA3_ = 0.0065, *p*_DG_ = 0.0023). However, the UA treatment reversed this change in CA3 and DG. Interestingly, in the CA1 region of the rat hippocampus, NPY was mainly expressed in the cytoplasm in normal rats, while NPY was mainly observed in synapses in the SE group. UA20 treatment increased the expression of NPY in synapses in CA1 without inducing the number of NPY^+^ cells (*p* = 0.3266) (Figures 10A–D). In summary, our results demonstrated that UA had a protective effect on GABAergic neurons during SE.

## Discussion

Neuroinflammation commonly occurs in human epileptogenic brain regions and animal epilepsy models (Vezzani et al., 2019). In inflammatory response, pro-inflammatory factors are biosynthesized and released from brain-resident cells, including activated microglia and neurons. Protracted and intensive neuroinflammatory response leads to cellular dysfunction during seizures. In addition, elevated oxidative stress is strictly associated with neuroinflammation, resulting in a mutual promotion. The acute increased mitochondrial oxidative stress following protracted seizure causes oxidative damage to mtDNA (Waldbaum and Patel, 2010). Furthermore, mitochondrial dysfunction is involved in the excitotoxicity induction of neuronal death (Green and Reed, 1998) and contributes to seizure-induced hippocampal cell loss and cognitive defects (Waldbaum and Patel, 2010; Kovac et al., 2017; Guo et al., 2020). Herein, we clarified the notable anti-seizure and neuroprotective properties of UA in pilocarpine-induced epileptic rats. In line with previous studies, the expression levels of pro-inflammatory factors IL-1β and TNF-α were remarkably increased by SE induction. Microglia was also excessively activated in SE-induced rats (Rizzi et al., 2003; Deng et al., 2019). In contrast, the UA treatment suppressed microglial activation by largely restraining the expressions of IL-1β and TNF-α. In addition, UA showed antioxidative stress activity in seizures, represented by decreased 8-OHdG and MDA levels. Indeed, similar results were also obtained in an MPTP-induced PD model with UA treatment in a previous study (Rai et al., 2016). Although UA100 treatment showed anti-inflammation and antioxidative effects at 24 h post-SE, the inhibitory effect of UA100 treatment on microglia was markedly stronger than the UA20 treatment. The expression levels of IL-1β and TNF-α were higher than in the UA20 treatment. Meanwhile, the UA100 treatment hardly protected hippocampal neurons against the damage induced by SE. Conversely, UA20 treatment remarkably ameliorated seizures and protected neurons. It is supposed that the anti-inflammation and neuroprotection effects of UA100 treatment vary due to the cytotoxicity of UA100 treatment at different time points. Furthermore, UA improved the cognitive impairment, which suggested that UA is a promising candidate for epileptic sequelae treatment. Therefore, our results implied the role of the anti-inflammatory and antioxidative effects of UA on epileptogenesis and ameliorating cognitive dysfunction induced by epilepsy.

Insufficient GABA inhibition triggers epilepsy. Massive loss of hilar GABAergic interneurons promotes hippocampus hyperexcitability, which augments seizures. Cognitive function is also impaired due to the persisting synaptic transmission (Buckmaster et al., 2017). In this study, we found that UA treatment reversed the reduction of positive GABAergic cells induced by SE, such as SS, PV and NPY positive cells. Indeed, PV^+^, NPY^+^, and SS^+^ interneurons are critical in maintaining normal cognitive function and neurogenesis. Neuron excitatory activity is also inhibited by those interneurons in the hippocampus (Kazmi et al., 2012). Furthermore, NPY dynamically regulates neurogenesis of adults in the subventricular zone (SVZ) and subgranular zone (SGZ). Therefore, UA improves cognitive function, possibly relying on GABAergic regulation. Interestingly, TNF-α downregulates GABAARs on the cell surface and participates in synaptic strength modulation during learning and memory (Pribiag and Stellwagen, 2013). Elevated expression of TNF-α was found in SE-induced rats in this study. UA treatment repressed expression of TNF-α, suggesting that UA may improve cognition by reducing TNF-α during SE.

Correct and ordered cell migration plays an important role in neurogenesis. The migration can be ravaged by external insults such as epilepsy, leading to severe neurodevelopmental disorders (Stouffer et al., 2016). Dentate neurogenesis, synaptic reorganization, and the appearance of granule cells are involved in ectopic location (Parent et al., 1997). In normal rats, neuronal precursors reside in the SGZ of the DG region, proliferating and migrating continuously to the granulosa cell layer (Kuhn et al., 1996). Prolonged seizure dysregulates the migration of DG progenitor cells (Parent et al., 2006; Gong et al., 2007a; Walter et al., 2007). Aberrant neurogenesis and ectopic migration drive DG toward the Hilar region after SE induction (Hung et al., 2013). Our results showed that DCX-marked newborn neurons were reduced, and Prox1-marked neuronal progenitors were migration driving DG toward the Hilar region in the SE model rats. These alterations may relate to the inflammatory and microglia activation. Furthermore, reelin is involved in modulating progenitor migration in DG. Reelin insufficiency potentially contributes to ectopic migration and aberrant integration of newborn neurons during the seizure (Gong et al., 2007b). In adult rats, reelin is mainly expressed in hilar inhibitory interneuron subsets, which are vulnerable to epileptogenic insults (Ishii et al., 2016). Considering the role of neuroinflammation in ectopic migration of neural progenitor cells in chronic epilepsy, UA may repress the formation of hilar-ectopic Prox1 positive cells based on the anti-inflammation effect. In addition, our research provides evidence that UA protects GABAergic interneurons from damage. It is supposed that UA ameliorates the ectopic migration of neuronal precursors by inhibiting seizure-induced GABAergic damage and inducing reelin expression.

In summary, UA showed a protective effect against lithium-pilocarpine-induced SE. Seizure-induced neurodegeneration and behavioral abnormalities were ameliorated by UA treatment. UA alleviated seizures and protected neurons *via* repressing inflammation and oxidation. In addition, UA protected GABAergic interneurons from damage and ameliorated abnormal neuronal ectopic migration induced by seizures. Neurogenesis was also enhanced by UA treatment. In the present study, we adopted a combination of preventive administration and therapeutic administration. Even though preventive administration has limitations in acute epileptic seizures and in clinic, post-epilepsy drug treatment is still meaningful for the prevention of chronic epilepsy development and cognitive impairment. Since epileptic seizures are mainly caused by the abnormal electrical activity of neurons (Thijs et al., 2019), the effect of UA on neuronal electrical activity during epilepsy needs to be further elucidated. In addition, the molecular mechanisms of UA function also require a more detailed explanation. Our study provides scientific evidence that UA is a potential drug candidate for epilepsy prevention and treatment.

## Data Availability

The raw data supporting the conclusion of this article will be made available by the authors without undue reservation.

## Abbreviations

TLE, temporal lobe epilepsy; SE, status epilepticus; UA, ursolic acid; ASDs, anti-seizure drugs; ROS, reactive oxygen species; AD, Alzheimer’s disease; PD, Parkinson’s disease; GABA-T, GABA transaminase; VPA, valproic acid; EEG, encephalographic; OFT, open-field test; PFA, paraformaldehyde; ELISA, enzyme-linked immunosorbent assay; SS, somatostatin; PV, parvalbumin; NPY, neuropeptide Y; DCX, doublecortin; 8-OHdG, 8-hydroxy-2-deoxyguanosine; MDA, malondialdehyde; SVZ, subventricular zone; SGZ, subgranular zone; DEGs, differentially expressed genes.
