# Supplementary figures and images for: Ursolic Acid Protects Neurons in Temporal Lobe Epilepsy and Cognitive Impairment by Repressing Inflammation and Oxidation
Source: Front Pharmacol. 2022 May 16;13:877898. doi: 10.3389/fphar.2022.877898 (PMC9169096; doi:10.3389/fphar.2022.877898)

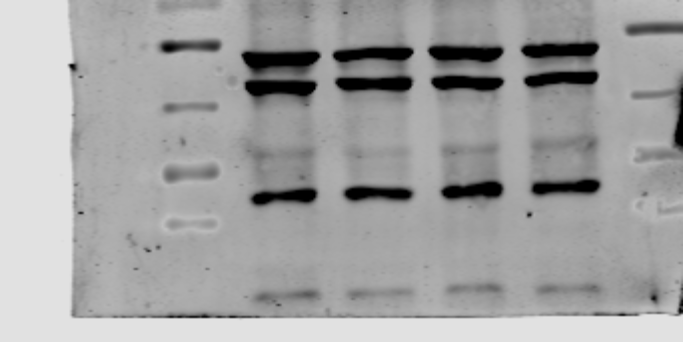

Supplement: Supplementary file 1 [file Image3.TIF]

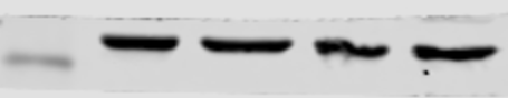

Supplement: Supplementary file 2 [file Image4.TIF]

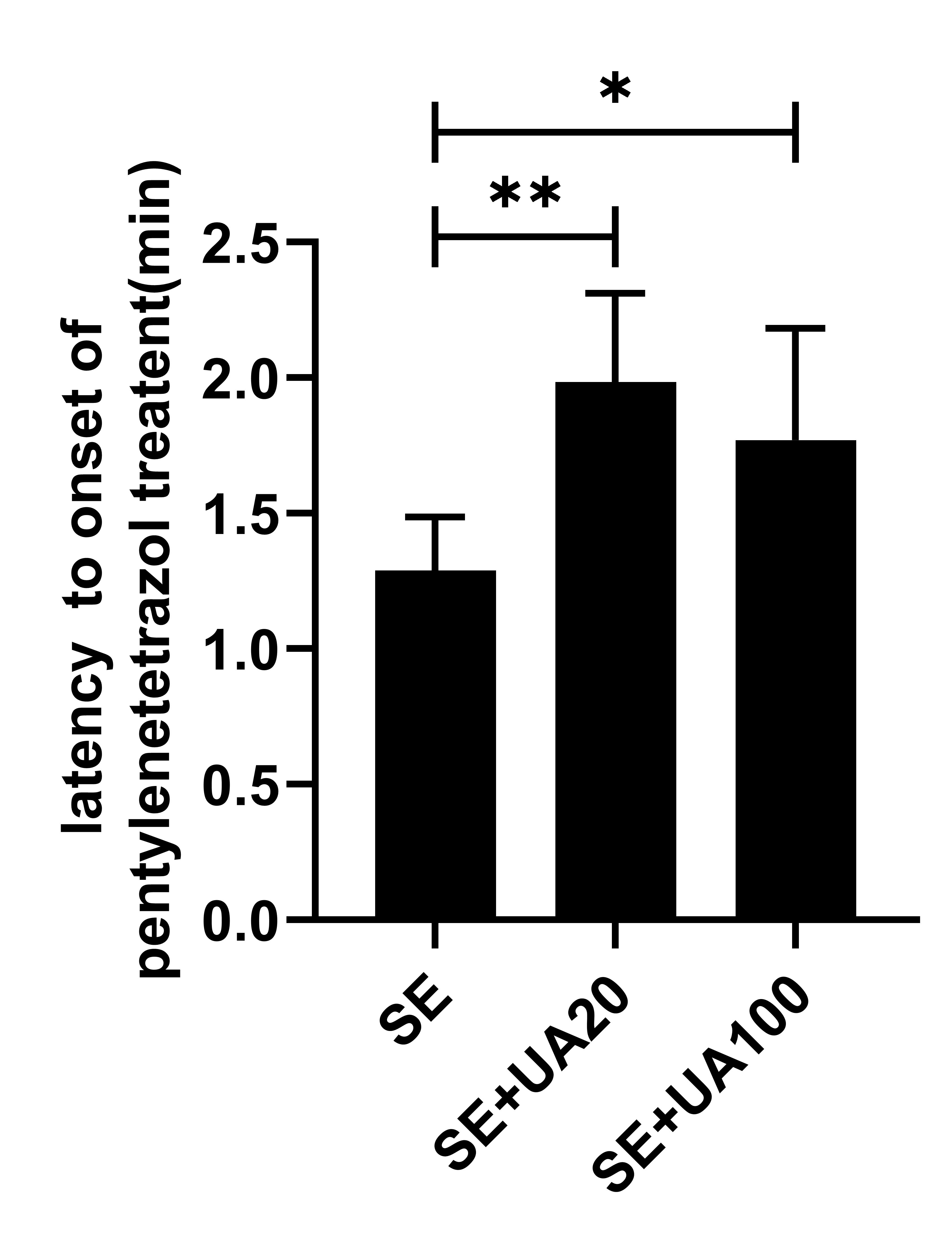

Supplement: Supplementary file 3 [file Image2.TIF]

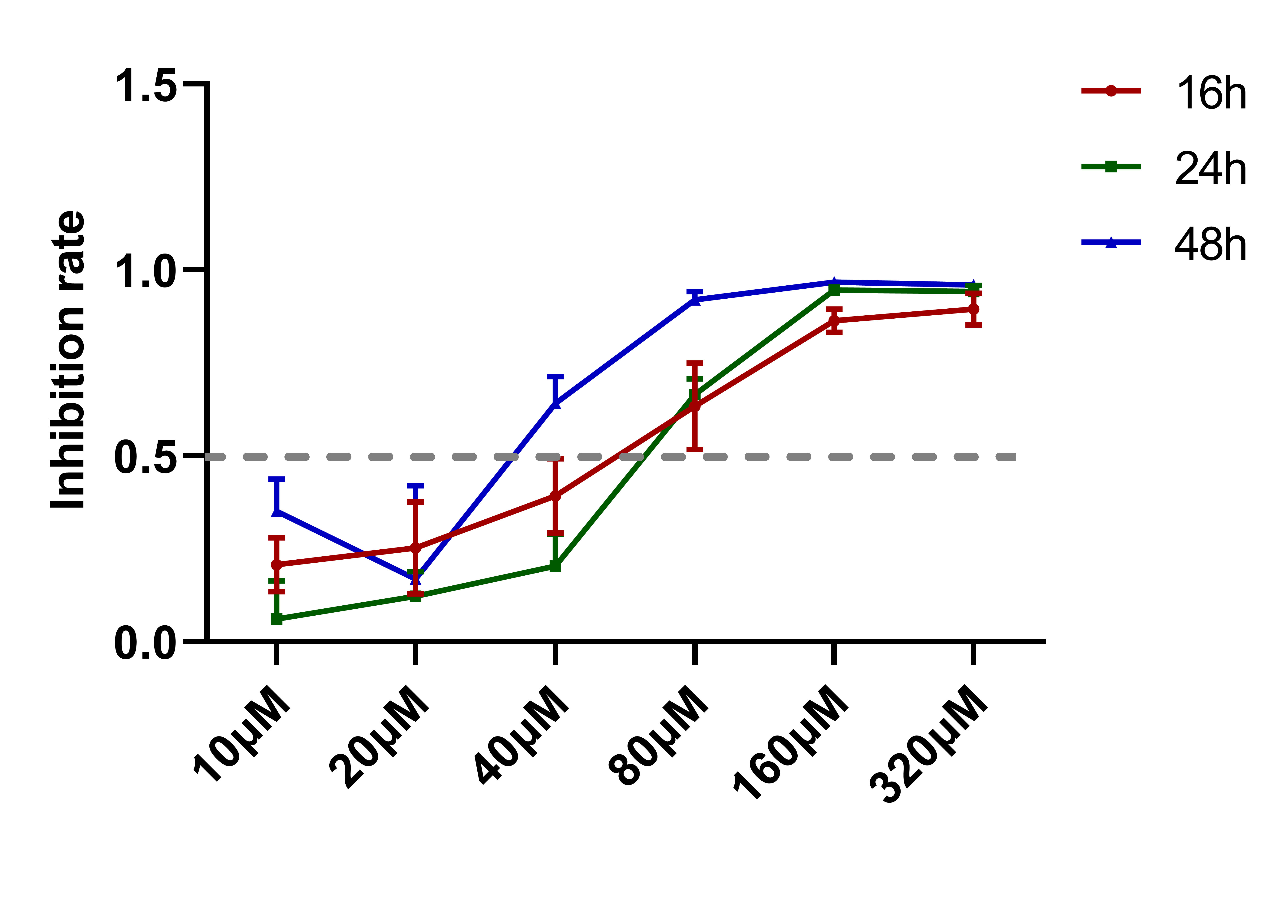

Supplement: Supplementary file 4 [file Image1.TIF]
