# Supplementary material for: Ursolic Acid Protects Neurons in Temporal Lobe Epilepsy and Cognitive Impairment by Repressing Inflammation and Oxidation
Source: Front Pharmacol. 2022 May 16;13:877898. doi: 10.3389/fphar.2022.877898 (PMC9169096; doi:10.3389/fphar.2022.877898)
Supplement: Supplementary file 5 [file DataSheet1.docx]

Supplementary Material

## Supplementary Figures

**
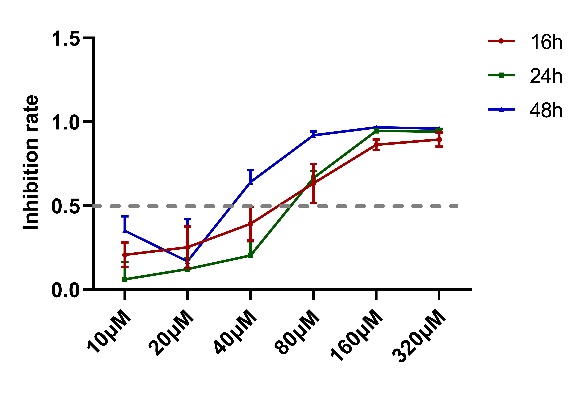
**

**Figure S1.** Inhibition rate of UA on Bv-2 cells at different doses.

**
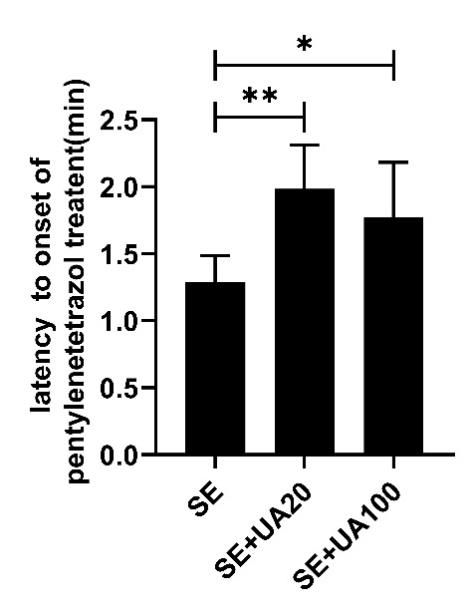
**

**Figure S2.** Latency of seizure occurrence induced by pentylenetetrazol.


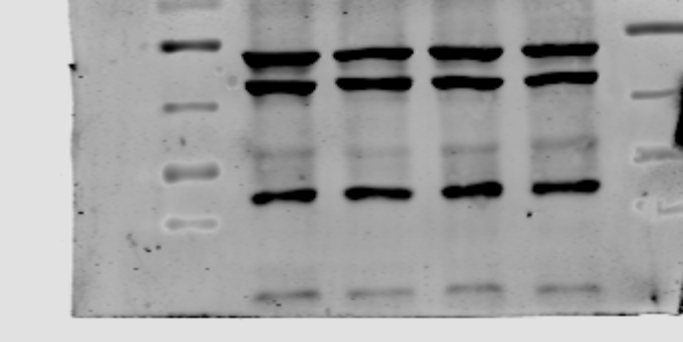


**Figure S3**. Raw images of Western blot for oxidative phosphorylation (OXPHOS) enzyme complexes of mitochondria. The lanes from left to right were marker, NC group, SE group, SE+U20 group, SE+UA100 group and marker. The sizes of marker bans from top to bottom were 55 KD, 40 KD, 35 KD and 20 KD, respectively.


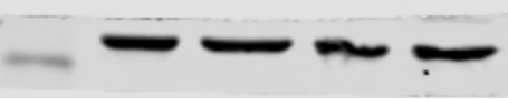


**Figure S4**. Raw images of Western blot for β-actin. The lanes from left to right were marker, NC group, SE group, SE+U20 group and SE+UA100 group. Marker: 40 KD.
